# Differential methylation in *EGLN1* associates with blood oxygen saturation and plasma protein levels in high-altitude pulmonary edema

**DOI:** 10.1186/s13148-022-01338-z

**Published:** 2022-09-30

**Authors:** Kavita Sharma, Aastha Mishra, Himanshu Singh, Tashi Thinlas, M. A. Qadar Pasha

**Affiliations:** 1 Genomics and Molecular Medicine, CSIR-Institute of Genomics and Integrative Biology, Delhi, India; 2 SNM Hospital, Leh, Ladakh India; 3 Institute of Hypoxia Research, Hypobaric Hypoxia Society, Delhi, New Delhi 110067 India

**Keywords:** Hypobaric hypoxia, Prolyl hydroxylases, DNA methylations, Prolyl hydroxylase domain protein 2, Factor inhibiting HIF-1α, High altitude

## Abstract

**Background:**

High-altitude (HA, 2500 m) hypoxic exposure evokes a multitude of physiological processes. The hypoxia-sensing genes though influence transcriptional output in disease susceptibility; the exact regulatory mechanisms remain undetermined in high-altitude pulmonary edema (HAPE). Here, we investigated the differential DNA methylation distribution in the two genes encoding the oxygen-sensing HIF-prolyl hydroxylases, prolyl hydroxylase domain protein 2 (PHD2) and factor inhibiting HIF-1α and the consequent contributions to the HAPE pathophysiology.

**Methods:**

Deep sequencing of the sodium bisulfite converted DNA segments of the two genes, *Egl nine homolog 1 (EGLN1)* and *Hypoxia Inducible Factor 1 Subunit Alpha Inhibitor (HIF1AN),* was conducted to analyze the differential methylation distribution in three study groups, namely HAPE-patients (HAPE-p), HAPE-free sojourners (HAPE-f) and healthy HA natives (HLs). HAPE-p and HAPE-f were permanent residents of low altitude (< 200 m) of North India who traveled to Leh (3500 m), India, and were recruited through Sonam Norboo Memorial (SNM) hospital, Leh. HLs were permanent residents of altitudes at and above 3500 m. In addition to the high resolution, bisulfite converted DNA sequencing, gene expression of *EGLN1* and *HIF1AN* and their plasma protein levels were estimated.

**Results:**

A significantly lower methylation distribution of CpG sites was observed in *EGLN1* and higher in *HIF1AN* (*P* < 0.01) in HAPE-p compared to the two control groups, HAPE-f and HLs. Of note, differential methylation distribution of a few CpG sites, 231,556,748, 231,556,804, 231,556,881, 231,557,317 and 231,557,329, in *EGLN1* were significantly associated with the risk of HAPE (OR = 4.79–10.29; *P* = 0.048–004). Overall, the methylation percentage in *EGLN1* correlated with upregulated plasma PHD2 levels (*R* = − 0.36, *P* = 0.002) and decreased peripheral blood oxygen saturation (SpO_2_) levels (*R* = 0.34, *P* = 0.004). We also identified a few regulatory SNPs in the DNA methylation region of *EGLN1* covering chr1:231,556,683–231,558,443 suggestive of the functional role of differential methylation distribution of these CpG sites in the regulation of the genes and consequently in the HIF-1α signaling.

**Conclusions:**

Significantly lower methylation distribution in *EGLN1* and the consequent physiological influences annotated its functional epigenetic relevance in the HAPE pathophysiology.

**Supplementary Information:**

The online version contains supplementary material available at 10.1186/s13148-022-01338-z.

## Background

Oxygen is vital for all living organisms as their evolution relies on the body’s homeostasis mechanisms for the efficiency of the energy-generating processes [1]. Oxygen also acts as a developmental morphogen influencing the differentiation of the progenitor cells [2]. These cellular differentiations are primarily driven through oxygen-modulated epigenetic modifying enzymes such as the Ten-Eleven-Translocation family of dioxygenases (TETs) and DNA methyltransferases (DNMTs) [3, 4]. Any deviation in the oxygen supply chain can be detrimental to human beings and manifests various cardiovascular pathophysiologies [5]. Nonetheless, the human body has a remarkable ability to sense and adapt to the changes in oxygen availability. These adaptations are primarily carried by the hypoxia-inducible factor-1α (HIF-1α) that transactivates several genes in response to oxygen fluctuations [6]. Along with HIF, the two oxygen sensors, Prolyl hydroxylase domain protein 2 (PHD2) and Factor inhibiting HIF-1α (FIH-1), play pivotal roles in the regulation of the hypoxia signaling pathway [7]. They utilize molecular oxygen as a co-substrate to accelerate hydroxylation of the HIF-1α subunit leading to its degradation and inactivation under the normoxic condition [7]. The gene names encoding PHD2 and FIH-1 are *Egl nine homolog 1 (EGLN1)* and *Hypoxia Inducible Factor 1 Subunit Alpha Inhibitor (HIF1AN), respectively.* The HIF-1α pathway being cardinal to human life, attained maximum attention to understand the underlying physiological processes under hypobaric hypoxia conditions prevalent at high altitude (HA), 2500 m above sea level [8]. Despite the interesting insights on hypoxia as a driving force of numerous genetic adaptations and maladaptation, our current knowledge on epigenetic modifications of the oxygen-sensing genes remains inadequate. The physiological response to hypoxia is impacted by both genetic and epigenetic mechanisms [8, 9]. The methylation of hypoxia response element (HRE) recognized by HIF is poorly understood, but it can potentially impact the HIF transactivation of its target genes [10, 11]. Additionally, the presence of CpG islands in numerous HIF signaling genes emphasizes the overall influence of epigenetics on the hypoxia response of the human body [12]. DNA methylation, one of the major epigenetic modifications regulating gene expression, is the mediator of crosstalk between genes and the environment [13]. It occurs at the 5′-cytosine position of CpG dinucleotide sites located at the CpG rich regions known as CpG islands, primarily present in the promoters of the genes. Both *EGLN1* and *HIF1AN* contain CpG islands, implicating a probable role of epigenetics in regulating their respective gene expression and subsequently to the HIF signaling.

PHD2 works by hydroxylating two proline residues in the oxygen-dependent degradation domain of HIF-1α so that it is recognized by the Von Hippel–Lindau protein ubiquitin ligase machinery for its proteasomal degradation [14]. In contrast, FIH-1 hydroxylates the asparagine residue in the transactivation domain of the HIF-1α subunit to block the binding of HIF-1α with co-activators p300/CREB-binding protein inhibiting its transcriptional activity [15]. Since oxygen is critical for the survival of both hydroxylases, the low oxygen condition or hypoxia suppresses their catalytic activities, stabilizing the HIF-1α subunit. Our prior work had identified the differential distribution of *EGLN1* polymorphisms and altered transcription factors on respective loci highlighting the genetic role of *EGLN1* on pathophysiological regulations in high-altitude pulmonary edema (HAPE) [16, 17]. This acute and severe HA illness occurs in unacclimatized individuals rapidly exposed to HA. Mode of ascent, altitude, speed and individual susceptibility are the most critical determinants for the occurrence of HAPE [18]. In continuation with our pursuit of *EGLN1* regulation at HA and its importance in HAPE pathophysiology, the present study investigated the epigenetic roles in differential DNA methylation distributions of *EGLN1* and *HIF1AN* and their associations with the clinical outcome. The study performed targeted deep sequencing of the two genes to evaluate the methylation percentage in their respective CpG islands in the three study groups, namely HAPE-patients (HAPE-p) and the two healthy control groups HAPE-free sojourners (HAPE-f) and healthy HA natives (HLs). HAPE-p and HAPE-f were permanent residents of low altitude (< 200 m) of North India and were of Indo-Aryan ethnicity; both groups visited HA. HAPE-p were sojourners who suffered the disorder upon exposure to HA. HAPE-f were the healthy subjects who visited HA under similar conditions and carried out routine strenuous physical activities but did not suffer from the disorder. HLs were permanent residents of altitudes at and above 3500 m for many generations of Tibeto-Burman ethnicity [19]. Subsequently, a correlation of the methylation percentage with respective protein expressions and the peripheral blood oxygen saturation (SpO_2_) levels was performed to understand the functional consequences of these epigenetic modifications in the disease pathophysiology. The DNA methylation investigation of the two genes provided interesting insights into the engagement of CpG sites in regulating the two genes and consequently in the HIF-1α signaling at HA.

## Results

### Clinical parameters reveal low blood peripheral oxygen saturation levels in HAPE

Clinical parameters differed significantly in HAPE patients compared to the control groups, i.e., HAPE-f and HLs (Table 1). Of note, in patients, SpO_2_ decreased significantly as compared to the two control groups (*P* < 0.0001, Additional file 1: Fig. 1); whereas it remained comparable in the two control groups, i.e., HAPE-f and HLs (*P* > 0.05).

**Table 1:** Clinical characteristics of the study groups, namely HAPE-p, HAPE-f and HLs

Clinical characteristics	HAPE-p (*n* = 32)	HAPE-f (*n* = 32)	HLs (*n* = 32)	*P* values		
				HAPE-p versus HAPE-f	HAPE-p versus HLs	HAPE-f versus HLs
Gender						
Male	28	32	28			
Female	4	0	4	NA	> 0.05	NA
Age, years	33.6 ± 11.8	24.2 ± 4.3	44.2 ± 8.4	0.008	< 0.001	< 0.0001
BMI, kg/m^2^	28.2 ± 6.4	19.9 ± 2.5	21.9 ± 2.5	< 0.0001	< 0.001	< 0.01
SBP, mmHg	131.2 ± 12.2	121.7 ± 9.4	116 ± 8.4	0.008	< 0.0001	0.020
DBP, mmHg	88.1 ± 7.7	81.0 ± 5.6	69.9 ± 4.8	0.002	< 0.0001	< 0.0001
MAP, mmHg	102.5 ± 11.3	94.6 ± 6.0	85.3 ± 5.0	< 0.001	< 0.0001	< 0.0001
SpO_2_ levels, %	67.7 ± 9.7	91.8 ± 4.4	92.3 ± 3.5	< 0.0001	< 0.0001	NS

Data are presented as mean ± SD and are compared by unpaired student’s t test.

*DBP* Diastolic blood pressure, *MAP* Mean arterial pressure, *n* Number of samples, *SpO*_*2*_ Peripheral blood oxygen saturation, *SBP* Systolic blood pressure, *NS* Non-significant, *NA* Not-applicable

### Gene expression and plasma protein level

**Fig. 1:**
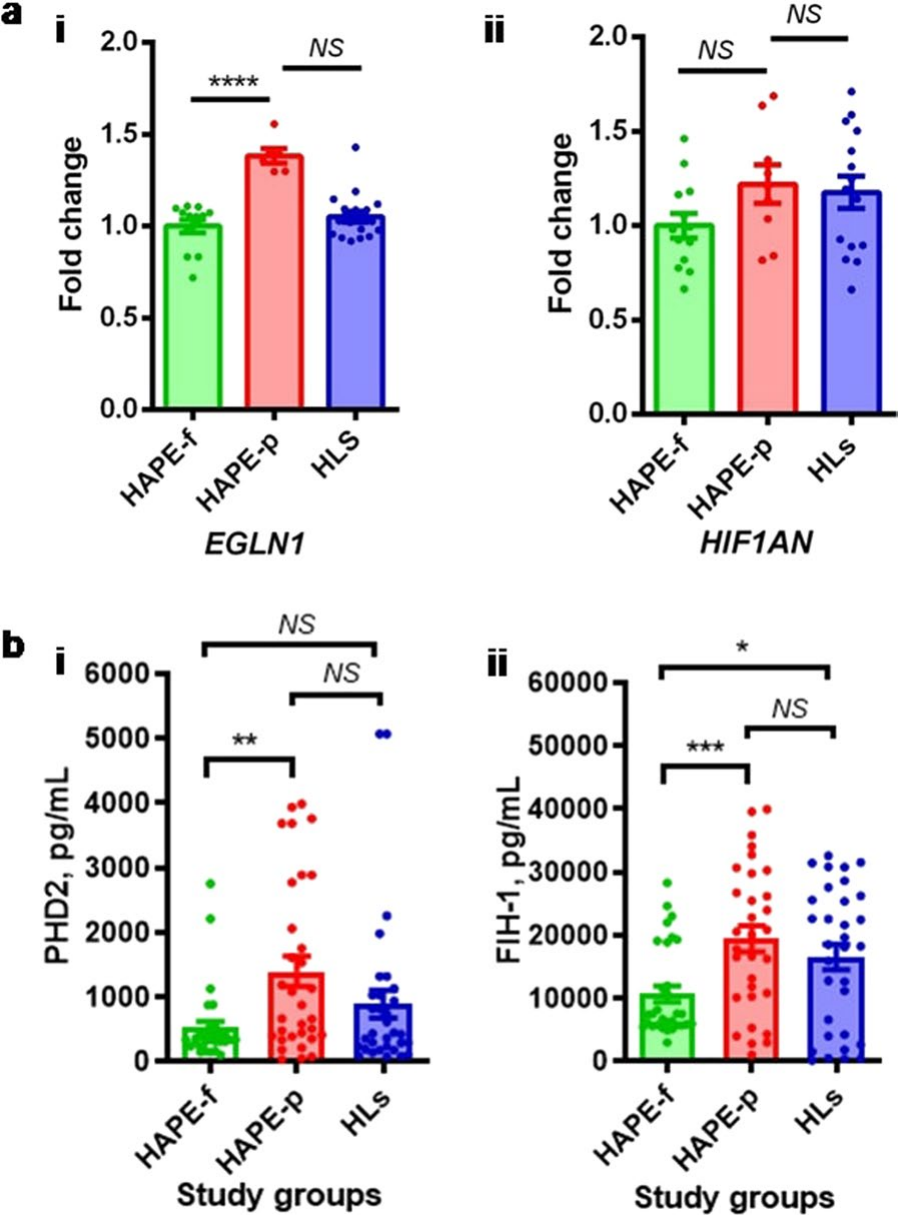
Gene expression and biochemical levels in the three study groups, HAPE-p, HAPE-f and HLs. **a** The relative expression as evaluated by real-time PCR, **(i)** *EGLN1* and **(ii)** *HIF1AN*. Values are expressed as fold-change in the HAPE-p and HLs with respect to HAPE-f. **b** Plasma levels, pg/mL, **(i)** PHD2 and (**ii)** FIH-1. Error bar represents the standard error of the mean (SEM); the statistical analysis was done using Student’s *t* test. Significance was maintained at *P* ≤ 0.05. EGLN1: Egl nine homolog 1; FIH-1: Factor inhibiting HIF-1α; HIF1AN: Hypoxia Inducible Factor 1 Subunit Alpha Inhibitor; PHD2: Prolyl hydroxylase domain protein 2; HAPE-p: HAPE-patients; HAPE-f: HAPE free controls; HLs: healthy highlanders; *P < 0.05; **P < 0.01; ***P < 0.00; ****P < 0.0001; *NS* Not significant

The *EGLN1* mRNA expression was1.38 fold higher in HAPE-p compared to HAPE-f (*P* < 0.0001; Fig. 1ai). However, no such significance was observed with *HIF1AN* gene expression (Fig. 1aii). In the case of protein expression, the plasma levels of both PHD2 and FIH-1 were significantly upregulated in HAPE-p compared to the HAPE-f (*P* < 0.001; Fig. 1 b i & ii).

### Targeted gene methylation profiling reveals differential patterns

**Fig. 2:**
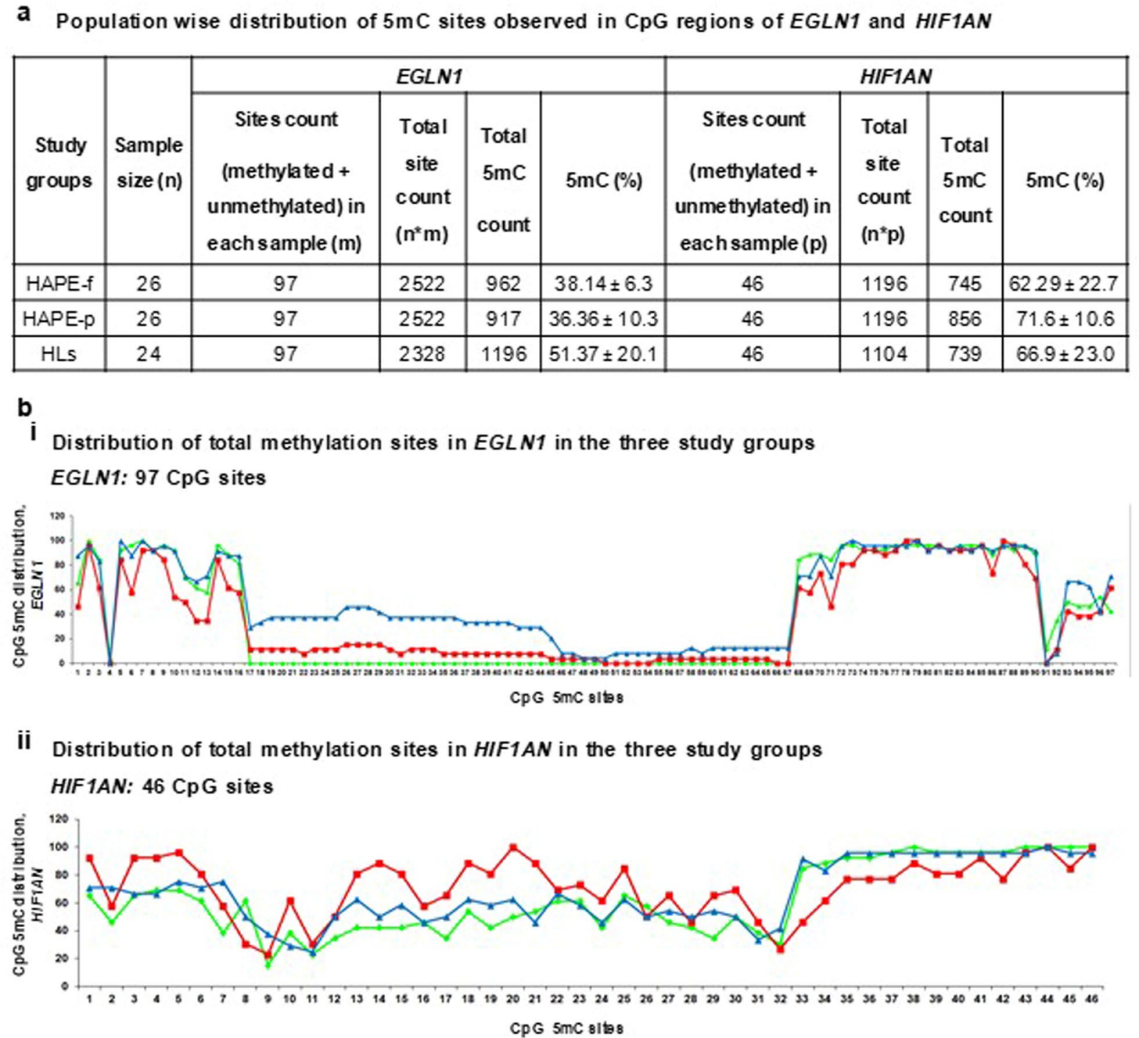
CpG dinucleotide sites distribution in *EGLN1* and *HIF1AN.* **a** Methylation distribution of CpG sites of *EGLN1* and *HIF1AN* in the three study groups i.e., HAPE-f, HAPE-p and HLs. **b** Line graph showing the percentage distribution of each site **(i)** *EGLN1* with respect to 97 CpG sites and **(ii)** *HIF1AN* with respect to 46 CpG sites. *EGLN1*: Prolyl hydroxylase domain protein 2; *HIF1AN*: Factor inhibiting HIF-1α; 5mC: 5 methylcytosine; %: percentage; HAPE-p: HAPE-patients; HAPE-f: HAPE free controls; HLs: healthy highlanders. Green color–-: HAPE-f; Red color–-: HAPE-p; Blue color–-: HLs

**Fig. 3:**
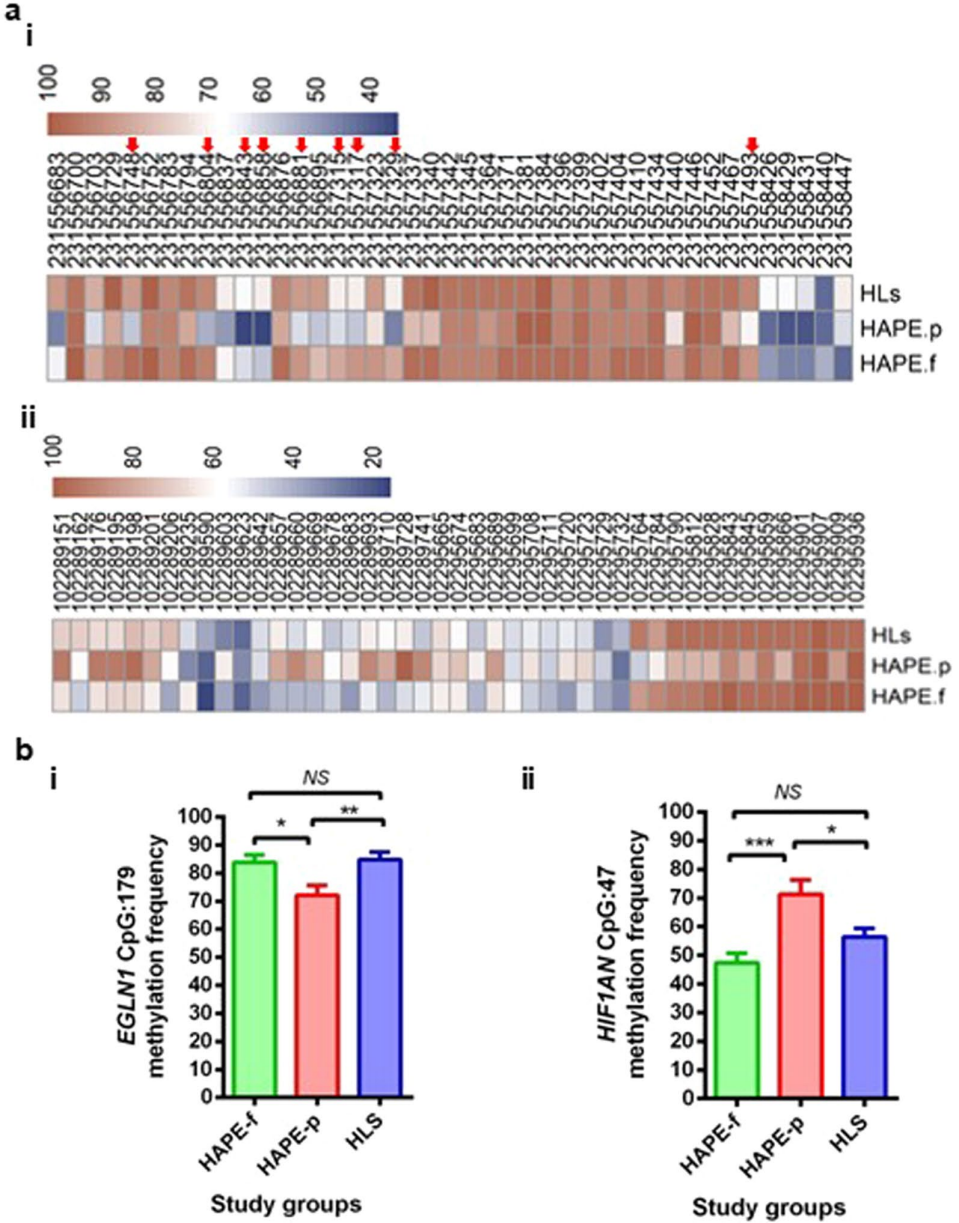
Methylation Frequency of CpG sites that showed differential distribution in the three study groups, i.e., HAPE-p, HAPE-f and HLs. **a** Heat-map shows the qualitative distribution of **(i)** 43 CpG methylation sites in *EGLN1,* and **(ii)** 45 CpG methylation sites in *HIF1AN*. **b** Graphs with the quantitative distribution of methylation frequency in the three study groups for **(i)** 43 CpG methylation sites in *EGLN1,* and **(ii)** 45 CpG methylation sites in *HIF1AN.* Red arrow symbol represents those CpG sites in EGLN1 gene that were differentially methylated in the study groups. *EGLN1*: Prolyl hydroxylase domain protein 2; *HIF1AN*: Factor inhibiting HIF-1α; HAPE-p: HAPE-patients; HAPE-f: HAPE free controls; HLs: healthy highlanders

After the extensive quality checks of libraries for 32 samples in each group, only 26 HAPE-p, 26 HAPE-f and 24 HLs samples proceeded for deep sequencing and further analysis. Sequential analysis was performed starting with the entire CpG regions of the two genes, *EGLN1* and *HIF1AN*, to shorter regions and finally to specific sites. The dot plot of CpG methylation in *EGLN1* in the three study groups revealed 97 CpG sites in *EGLN1* CpG island 179 and 46 CpG sites in *HIF1AN* CpG island 47 (Additional file 1: Figs. 2 and 3). A population-wise distribution of 5mC sites observed in the CpG region for each gene was calculated as the ratio of the total 5mC (methylated) count and the entire site (methylated and unmethylated) count for each study group (Fig. 2a). The differential methylation of CpG sites in *EGLN1* in the three groups mostly occurred at the two peripheries of the CpG island, while a substantial part of the central region remained unmethylated. *HIF1AN* CpG island, on the other hand, presented a rather scattered distribution (Fig. 2bi & ii). The cumulative methylation percentage of CpG sites in *EGLN1* was 36.3 ± 10.6 in HAPE-p as compared to 38.1 ± 6.3 in HAPE-f (*P* ≤ 0.05) and 51.4 ± 20.1 in HLs (Fig. 2a & Additional file 1: Table 1). The cumulative methylation percentage of CpG sites in *HIF1AN* was 71.6 ± 10.6 in HAPE-p compared to 62.3 ± 22.7 in HAPE-f and 66.9 ± 23 in HLs (Fig. 2a & Additional file 1: Table 1). As shown in the heat map, a detailed analysis of each CpG site in all the samples showed that only 43 CpG sites out of 97 in *EGLN1* and 45 CpG sites out of 46 in *HIF1AN* demonstrated the differential distribution (Fig. 3ai & ii). The cumulative methylation percentage from these selected CpG sites further improved the significance of differential distribution in the three study groups. The methylation percentage of the CpG island of *EGLN1* in HAPE-p was significantly decreased compared to the two control groups (*P* < 0.01; Fig. 3bi). However, for *HIF1AN,* the methylation percentage in HAPE-p was significantly increased compared to the two controls (*P* < 0.01, Fig. 3bii).

### Altered DNA methylation correlates with plasma PHD2 and blood peripheral oxygen saturation level

**Fig. 4:**
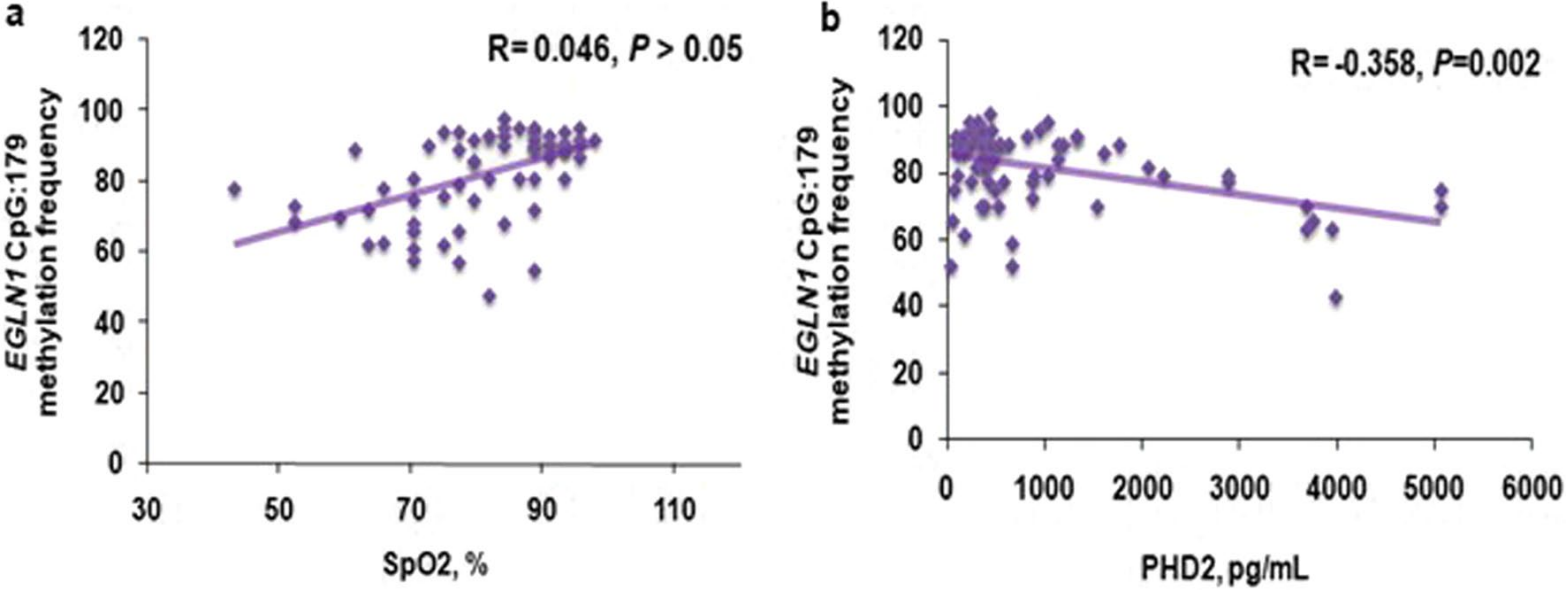
*EGLN1* correlation analysis. **a** Scatter plot for the correlation analyses between methylation percentage distribution and SpO_2_ level, %. **b** Scatter plot for the correlation analyses between methylation percentage distribution and plasma PHD2 level, pg/mL. Significance was maintained at *P* ≤ 0.05. *P* values and R were obtained by bivariate and Pearson’s correlation analysis. EGLN1: Egl nine homolog 1; PHD2: Prolyl hydroxylase domain protein 2; SpO_2_: blood arterial oxygen saturation

The differential methylation percentage of *EGLN1* and *HIF1AN* in each individual of the three groups was examined with their respective SpO_2_ levels. A linear correlation existed between SpO_2_ level and the methylation percentage distribution in *EGLN1* (*R* = 0.34, *P* = 0.004; Fig. 4a). The differential methylation percentage of *EGLN1* and *HIF1AN* in each individual of the three groups was also examined with the respective gene and protein expressions. The increase in the translational expression of *EGLN1* in HAPE-p was in line with our observation of significantly lower methylation percentage distribution of *EGLN1* in the patients. Likewise, an inverse correlation of the plasma PHD2 levels with methylation percentage distribution was observed (*R* = − 0.36, *P* = 0.002; Fig. 4b).

### Potential specific CpG sites are susceptible to the disorder

The intuitive analysis of all CpG sites was performed using multivariate logistic regression that provided some interesting discernments. Of relevance, among these specific sites, we could not overlook nine CpG sites in *EGLN1* (231,556,748, 231,556,804, 231,556,843, 231,556,858, 231,556,881, 231,557,315, 231,557,317, 231,557,329, 231,557,493) that stood apart distinctly with differential distribution in our groups (Fig. 4c). As shown in Table 2, out of these sites, the CpG sites that differed significantly between HAPE-p and HAPE-f were 231,556,748 (OR = 8.33; *P* = 0.004), 231,556,804 (OR = 10.29; *P* = 0.011), 231,556,881 (OR = 4.79; *P* = 0.048), 231,557,317 (OR = 5.62; *P* = 0.022) and 231,557,329 (OR = 6.42; *P* = 0.006). Next, we performed a regression coefficient to show the association between these nine CpG sites and the plasma PHD2 levels in the three groups (Table 3). Two CpG sites, 231,556,748 and 231,557,315, were inversely associated with the plasma PHD2 level in HLs (*P* < 0.05). The in-depth analysis did not have any noteworthy results for CpG sites of *HIF1AN*.

**Table 2:** Methylation distribution and association analysis of differentially methylated CpG sites of *EGLN1* in HAPE-p, HAPE-f, and HLs

Differential methylation sites	HAPE-p (*n* = 26)	HLs (*n* = 24)	HAPE-f (*n* = 26)	HAPE-f versus HAPE-p			HLs versus HAPE-p			HAPE-f versus HLs		
	Distribution (%)			$\chi$^2^	P*	OR (95% CI)	$\chi$^2^	P*	OR (95% CI)	$\chi$^2^	P*	OR (95% CI)
231,556,748	57.70	87.50	96.20	7.07	0.004	8.33 (2.15–156.58)	4.96	0.178	5.13 (1.22–21.63)	1.14	0.919	8.57 (0.35–36.94)
231,556,804	53.80	91.70	92.30	7.80	0.011	10.29 (2–52.79)	7.19	0.027	9.43 (1.83–48.61)	0.01	0.836	1.09 (0.14–8.42)
231,556,843	34.60	66.70	61.50	3.68	0.15	3.02 (0.98–9.36)	4.94	0.129	3.78 (1.17–12.19)	0.14	0.35	0.8 (0.25–2.55)
231,556,858	34.60	70.80	57.70	2.73	0.075	2.58 (0.84–7.91)	6.24	0.96	4.59 (1.39–15.15)	0.93	0.811	0.56 (0.17–1.82)
231,556,881	61.50	87.50	88.50	4.55	0.048	4.79 (1.14–20.21)	4.01	0.214	4.38 (1.03–18.56)	0.01	0.08	1.1 (0.2–6.03)
231,557,315	61.50	70.80	84.60	3.33	0.012	3.44 (0.91–12.95)	0.48	0.779	1.52 (0.47–4.95)	1.34	0.475	2.27 (0.57–9.02)
231,557,317	57.70	70.80	88.50	5.58	0.022	5.62 (1.34–23.56)	0.93	0.585	1.78 (0.55–5.77)	2.28	0.413	3.16 (0.71–14.02)
231,557,329	46.20	70.80	84.60	7.68	0.006	6.42 (1.72–23.9)	3.04	0.081	2.83 (0.88–9.13)	1.34	0.569	2.27 (0.57–9.02)
231,557,493	69.20	91.70	88.50	2.70	0.041	3.41 (0.79–14.72)	3.47	0.54	4.89 (0.92–25.97)	0.14	0.992	0.7 (0.11–4.58)

P*, P values obtained after adjusting it with age and gender by multinomial logistic regression analysis using SPSS 16.0 software. The methylation distributions were compared by the χ^2^ test.

*n* Number of samples, (%) Percent distribution, *OR* Odds ratio, *CI* Confidence interval

**Table 3:** Differential methylation sites of *EGLN1* in relation to its protein (plasma PHD2) levels in the three study groups

S. No	Differential methylation sites	HAPE-f (*n* = 26)		HAPE-p (*n* = 26)		HLs (*n* = 24)	
		β	*P*	β	*P*	β	*P*
1	231,556,748	0.038	0.856	0.328	0.1	− 0.487	**0.018**
2	231,556,804	0.108	0.607	− 0.242	0.231	− 0.059	0.786
3	231,556,843	-0.291	0.156	− 0.033	0.87	− 0.131	0.55
4	231,556,858	-0.093	0.657	− 0.054	0.79	− 0.25	0.248
5	231,556,881	0.182	0.383	− 0.013	0.948	− 0.334	0.118
6	231,557,315	0.284	0.167	0.203	0.319	− 0.482	**0.019**
7	231,557,317	0.149	0.475	− 0.12	0.557	− 0.156	0.476
8	231,557,329	0.008	0.969	0.256	0.205	− 0.077	0.724
9	231,557,493	0.185	0.375	0.089	0.662	− 0.083	0.704

*P* values were obtained by bivariate correlation analysis and *β* values were obtained by linear regression analysis using SPSS 16.0. *β* Regression coefficient. *P* values in bold are statistically significant (*P* < 0.05)

### Single nucleotide polymorphisms in and around CpG sites

With the interesting results on the differentially and significantly distributed individual CpG sites in *EGLN1* in the three groups, we hypothesized that a single nucleotide polymorphism (SNP) in and around the differentially methylated sites might contribute to the regulation of the gene. We did in silico analysis to determine the presence of SNPs around these differentially methylated regions of *EGLN1* covering chr1:231,556,683–231,558,443 (Table 4). The SNP rs186996510, also known as 12C > G or Asp4Glu that lies upstream to the hypermethylated sites, chr1: 231,557,485 and 231,557,493, bears the transcription factor binding site for the transcription factor (TF) transforming growth factor-beta-induced factor homeobox 1 (TGIF1) (RegulomeDB Score, 2b). SNP rs12097901, also known as 380G > C or Cys127Ser, and rs61750991, also known as 471G > A or Gln157His, annotated to the two upstream regions of the hypomethylated sites, chr1: 231,556,843 and 231,556,858. While rs12097901 was associated with TF Forkhead Box B1 (FOXB1) (RegulomeDB Score, 3a), rs61750991 was associated with RNA Polymerase II Subunit A (POLR2A) and Fos Proto-Oncogene, AP-1 Transcription Factor Subunit (FOS), however, the association appeared poor with a RegulomeDB Score4.

**Table 4:** Functional annotation of the differentially distributed methylation sites in *EGLN1*

S. no	SNP rsID	Annotate site	Variant type	Allele	Amino Acid change	TF associated	RegulomeDB score	Methylation site	Methylation consequence
1	rs186996510	chr1:231,557,622	Missense	12C > G	Asp4Glu	TGIF1	2b	chr1:231,557,493	Hypermethylation
2	rs12097901	chr1:231,557,254	Missense	380G > C	Cys127Ser	FOXB1	3a	chr1:231,556,843	Hypomethylation
3	rs61750991	chr1:231,557,163	Missense	471G > A	Gln157His	No TF reported in literature	4	chr1:231,556,858	Hypomethylation

RegulomeDB v1.1 was used for annotation.

*TF* Transcription factor; 2b: TF binding + any motif + DNase Footprint + DNasepeak; 3a: TF binding + any motif + DNase peak; 4: TF binding + DNase peak

## Discussion

The present study identified the CpG islands with differentiated DNA methylation levels in the two oxygen-sensor prolyl hydroxylase genes, *EGLN1* and *HIF1AN*, regulating the HIF signaling pathway in the three study groups, HAPE-p, HAPE-f and HLs. Overall, the percentage of methylation distribution in the CpG island 179 of *EGLN1* was significantly lower and CpG island 47 of *HIF1AN* was significantly higher in HAPE-p compared to the two controls, HAPE-f and HLs. Of note, there were sites at a stretch that scarcely bore methylation and some that were ornately methylated. Moreover, in *EGLN1,* specific sites with methylation percentages were significantly lower in patients and were also associated with the risk of HAPE in the susceptible individuals. We could identify such distinct methylated CpG sites in *EGLN1* potentially adding to the HAPE risk. The elevated *EGLN1* expression and plasma PHD2 level in HAPE-p compared to its healthy counterparts suggested its deleterious role in HAPE. Importantly, our results demonstrated an inverse correlation between the plasma PHD2 level and methylation percentage of CpG sites in *EGLN1*. The methylation state in the CpG Island regulates the normal functioning of the gene [13]. Among the clinical parameters, SpO_2_ level was significantly decreased in patients, and the proportional correlation of methylation percentage of CpG sites in *EGNL1* with SpO_2_ level further gains significance in the manifestation of HA pathophysiology. These findings portray the functional consequences of the epigenetic modifications at HA. Similarly, *HIF1AN* expression and plasma FIH-1 level were upregulated in HAPE-p compared to HAPE-f and HLs. However, no such correlations of the methylation levels with the gene or protein expressions were observed for *HIF1AN*. Perhaps, the regulation of *HIF1AN* is not influenced by its methylation.

Several reports in the past and recent have clearly demonstrated the selection in the variants of *EGLN1* in the highland population around the world [20–22]. Bigham et al. [20] showed evidence of positive selection in *EGLN1* in both Tibetans and Andeans [20]. Further, our studies affirmed that the *EGLN1* risk alleles rs1538664A, rs479200T and rs480902C increased the *EGLN1* gene expression and were also associated with decreased SpO_2_ levels [17]. These alleles were explored for the additional contributions from the associated secondary molecules, especially the transcription factors (TFs) that may regulate the gene through the differential distribution of their variant alleles and the respective TFs in the healthy and susceptible subjects [16]. The study validated the specificity between a TF and allelic variants such as FUSRNA-binding protein (FUS) with rs1538664A, Rho GDP dissociation inhibitor 1 (RhoGDH1) with rs479200T, and hypoxia upregulated protein 1 (HYOU1) with rs480902C. Brutsaert et al. [21] found the increased frequency of an *EGLN1* causal variant, rs1769793 that enhances O_2_ delivery or use during exercise at altitude in the Peruvian Quechua population [21]. Xiang et al. [22] detected a significant association between rs186996510 and hemoglobin levels in Tibetans, suggesting that *EGLN1* contributes to the adaptively low hemoglobin level of Tibetans compared with acclimatized lowlanders at high altitudes [22]. Thus, the *EGLN1* genetics variants have been influential in affecting the physiological changes relevant to HA.

The present study explored the epigenetic factors, such as DNA methylation associated with this gene. The position of a CpG island studied in our study for differential DNA methylation does not coincide with the region of evolutionary selection seen in the *EGLN1* gene in most of the above studies; however, we still identified potential regulatory SNPs in the *EGLN1* methylation region covering chr1:231,556,683–231,558,443. For example, SNP rs12097901 annotated to the upstream regions of the CpG sites 231,556,840, 231,556,843, and 231,556,844 and SNPs rs186996510 upstream to the CpG sites 231,557,485 and 231,557,493 are reported to have been selected in the Tibetan population under the HA settings [23]. Further, SNP rs12097901 along with other coding region SNP rs186996510 may have adaptive benefits as both are associated with reduced hemoglobin phenotype characteristic of Tibetan adaptation to altitude [24]. The few other regulatory SNPs around the methylated region were also associated with transcription factors whose interactions might be influenced by the differential methylation, affecting the regulation of the gene and body physiology [25, 26]. A recent study outlined four one-carbon metabolism SNPs, *methylenetetrahydrofolate dehydrogenase 1* rs2236225, *Thymidylate Synthase* rs502396, *Folate Hydrolase 1* rs202676 and *glycine decarboxylase* rs10975681, that cumulatively explained 11.29% of the variation in average LINE-1 methylation among Andean Quechua population [27]. They also found that the number of years lived at HA was negatively associated with EPAS1 methylation and positively associated with LINE-1 methylation. Apart from HA, the epigenetic influence of *EGLNs* has been demonstrated as a potential marker for lung adenocarcinoma prognosis [28].

## Conclusions

Our study showed germane results for epigenetic modifications in *EGLN1* for HA adaptation and HAPE pathophysiology. It emphasized the differential methylation of *EGLN1* and *HIF1AN* in HAPE-p compared to the two control groups, i.e., HAPE-f and HLs. Based on the methylation frequency, HAPE-p showed a significantly lower methylation distribution in *EGLN1* but higher distribution in *HIF1AN*. Moreover, specific sites in *EGLN1* had significantly lower methylation distribution in HAPE and potentially added to the HAPE risk. We also identified potential regulatory SNPs in the methylation region of *EGLN1* that may get influenced by the differential methylation in the HA settings. The correlation studies of methylation distribution of *EGLN1* CpG island with plasma PHD2 level and SpO_2_ level further indicated the causal role in HA pathophysiology. Overall, our findings inclined towards a likely epigenetic regulatory role of *EGLN1* in the susceptibility to HAPE. Nonetheless, we realize that validation in greater sample sizes and various ethnicities would uphold the findings. Additional experimental studies determining the effects of each potential CpG dinucleotide site would authenticate its functional consequence. Further, non-matched patient groups and possible changes in cell populations driving some differential methylation patterns are a few other limitations of the study. The validation of our results in an in vitro setup may account for all the individual cofounders influencing epigenetic patterns that we plan to do in our future studies.

## Methods

### Study participants

Blood samples were obtained from subjects that were categorized into three well-defined groups: 1) HAPE-patients (HAPE-p) were sojourners who suffered the disorder upon exposure to HA; 2) HAPE-free sojourners (HAPE-f), who visited HA under similar conditions and carried out routine strenuous physical activities but did not suffer from the disorder and remained healthy, and 3) highland natives (HLs) were permanent residents of altitude at and above 3500 m for many generations with Tibeto-Burman ethnicity. HAPE-p and HAPE-f belonged to Indo-Aryan ethnicity and were permanent residents of low altitude (< 200 m) of North India who traveled to Leh, Ladakh, for reasons such as professional assignments, recreation, and adventure. Approximately 32 subjects from each group were recruited through Sonam Norboo Memorial (SNM) hospital, Leh (3500 m), Ladakh, India.

### Study approval

The human ethics committee of CSIR-Institute of Genomics and Integrative Biology, Delhi and SNM Hospital, Leh, Ladakh, India, approved the study for human subjects. All participants of the study whose identities were undisclosed gave written informed consent.

### Blood sample collection and clinical assessment

Eight milliliters of blood sample was collected from each subject in acid-citrate-dextrose anticoagulant. Blood samples of HAPE-p were drawn immediately after the diagnosis but before starting medication. Plasma and peripheral blood leukocytes were separated; the latter was processed for DNA extraction. Two ml of whole blood without anticoagulant was collected for RNA extraction. Plasma and RNA were stored at − 80 °C and DNA at − 20 °C. General clinical parameters for each subject were recorded. Diagnosis of HAPE was based on published clinical criteria [18]. The SpO_2_ level was measured by Finger-Pulse Oximeter 503 (Criticare Systems Inc, USA).

### Expression analysis of *EGLN1* and *HIF1AN*

#### Quantitative real-time PCR

Gene expression of *EGLN1* and *HIF1AN* was determined on 10 samples each of HAPE-p, HAPE-f, and HLs. Total RNA was extracted from a 2 ml whole blood sample aliquot without anticoagulant by TRI reagent RT blood (Molecular Research Centre, Cincinnati, USA). RNA quantity and quality were determined on a NanoDrop ND-1000 spectrophotometer, and integrity was checked on 1.5% agarose gel. Total RNA, 1.0 μg, was used to generate cDNA by EZ-first strand cDNA synthesis kit for reverse transcriptase-PCR (Biological Industries, BeitHaEmek, Israel). Real-time PCR was performed in triplicate with primers (Pearl Primer software; Additional file 1: Table 2) and SYBR Green PCR Master Mix on an ABI Prism 7300 Sequence Detection System (Applied Biosystems, Foster City, USA). The relative transcript quantity was calculated using the ΔΔC_t_ method against *18SrRNA* endogenous reference.

#### Estimation of plasma PHD2 and FIH-1 levels

Plasma PHD2 and FIH-1 levels were estimated by immunoassay kits (USCN Life Science, Wuhan, China) on a high-throughput SpectraMax plus384 Spectrophotometer (Molecular Devices, San Jose, USA).

### Targeted methylation pattern in *EGLN1* and *HIF1AN*

CpG islands in both the genes were confirmed by the UCSC genome browser (genome.ucsc.edu/) according to the February 2009 Human Genome Browser data. In silico study identified CpG islands, CpG179spanning the region chr1: 231,556,683–231,558,443 in *EGLN1* and CpG47 from the region chr10:102,289,150–102,296,000 in *HIF1AN*. Total methylation distribution and the actual percentage of methylation were quantified for *EGLN1* and *HIF1AN* concerning CpG sites.

#### Sodium Bi-sulfite conversion of DNA

Genomic DNA from whole blood was extracted from peripheral blood leukocytes using modified salting out procedure [17]. Quantification and quality check of DNA was carried out on a NanoDrop^TM^1000 Spectrophotometer (Thermo Scientific, USA). One μg of blood genomic DNA was converted to sodium bisulfite by EZ DNA Methylation-GoldTM Kit (Zymo Research, Irvine, USA). Briefly, DNA was bisulfite-converted for 16 h at 50 °C and subsequently desulfonated, washed, and eluted in 10 μl elution buffer. The method selectively converts cytosine (C) to uracil (U) without significant transformation of 5-methylcytosine (5mC) to thymine (T).

#### PCR Amplification of Bisulfite converted DNA

The bisulfite converted DNA of *EGLN1* and *HIF1AN* consisting CpG island region was PCR amplified using the bisulfite-conversion-based methylation PCR primers designed by the software methprimer (http://www.urogene.org/cgi-bin/methprimer/methprimer.cgi, Additional file 1: Table 3). PCR amplifications were achieved by Amplitaq gold DNA polymerase by using a range of varying annealing temperatures in a gradient thermocycler (Thermo Scientific, USA). The reaction conditions and amplicon size for each primer pair are given in Additional file 1: Table 3. PCR products were purified by QIAquick PCR columns (Qiagen, USA). The length and concentration of these amplicons were analyzed using an Agilent High Sensitivity DNA chip on Agilent 2100 bioanalyzer (Agilent Technologies, USA).

#### NGS library preparation and deep sequencing of Sodium Bisulfite converted amplicons

The Nextera DNA sample preparation kit from Illumina profiled the CpG islands in the MiSeq sequencing platform in thirty-one subjects, each from HAPE-p and HAPE-f groups and thirty-two subjects from the HLs group. According to the manufacturer's protocol, dual indexed libraries were generated (Illumina, San Diego, CA, USA). Each purified PCR product, one nanogram diluted, was used for library generation in a 96-well plate format. Tagmentation process that includes transposome-mediated simultaneous DNA fragmentation and adapter ligation was performed at 55 °C for 5 min. After the tagmentation, specific PCR primers were indexed for multiplex sequencing. Limited cycle-number PCR was performed to amplify the purified libraries using AMPure XP beads (Beckman-Coulter, Brea, CA, USA). Double-stranded libraries were quality checked for size and molarity determination on a high sensitivity DNA Agilent chip run on the Agilent 2100 Bioanalyzer (Agilent Technologies). Equimolar libraries were pooled in equal volumes for sequencing on the Illumina MiSeq benchtop sequencer as per the manufacturer’s protocol.

### Bi-sulfite sequence analysis

The variant calling algorithms counted the Cs and Ts at CpG sites in the reference sequences for quantitative digital methylation. Sequences were evaluated by mapping against the human reference genome using Illumina MiSeq system built-in Illumina MiSeq Reporter software. Bismark tool kit analyzed the bisulfite reads. The paired-end sequence reads were aligned to the in silico bisulfite-converted human reference genome hg19 in a strand-specific manner, not allowing any mismatches or multiple alignments. Deduplication was carried out and saved in the BAM format using deduplicate_bismark program. Methylation calls were extracted from the BAM files generated by deduplication, along with a short report detailing the calls. It generated mainly strand and context-specific cytosine output files and overall count report along with the HTML report. Genetic location is according to the February 2009 Human Genome Browser data [9]. Significance was maintained at ≤ 0.05.

### In silico identification of regulatory loci present within the CpG island

RegulomeDB ver 1.1 performed the functional annotation exercise within the differentially methylated regions. The identified regulatory SNPs associated with the methylated sites were annotated to understand their crucial role in gene regulation.

### Statistical analysis

The role of methylation in HAPE disease and health was evaluated by multivariate logistic regression analysis using SPSS 16.0 software. The study groups depicting phenotype were the dependent variable. While comparing, one group was considered as reference against the other groups. The reference group was labeled as 1, while the test group was labeled as 0, meaning cases/test. Since we wanted to test the significance of each methylated site in all the study groups to identify their association with the disease. Therefore, we tested individual methylation sites and considered each site as a fixed factor or categorical independent variable. To achieve this, we labeled subjects with no methylation for a site tested as 1 (reference) against the methylated site labeled 0. Finally, to derive the best-fitting and biologically reasonable model to describe the relationship between an outcome and a set of predictors, we adjusted the data with age and gender as covariates. The adjusted *P* value < 0.05 was considered significant. Odds ratio (OR) and 95% confidence interval (CI) were calculated. The SPSS16.0 and EPIINFO-6.0 software were used for analyses. Statistical analysis was performed using the standard two-tailed parametric Student’s *t* test. Multiple correlation analyses using Pearson’s correlation (r) values were performed for levels. The quantitative RT-PCR was analyzed by one-way analysis of variance. Values are represented as means ± standard deviation.

## Supplementary Information

**Additional file 1**. **Fig. S1** Levels of SpO_2_ % in HAPE-p, HAPE-f and HLs. **Fig. S2 **Dot plot of CpG methylation in *EGLN1 *in the three study groups, i.e., HAPE-p, HLs and HAPE-f. It revealed 97 CpG sites in *EGLN1 *CpG island 179. **Fig. S3 **Dot plot of CpG methylation in *HIF1AN *in the three study groups, i.e., HAPE-f, HAPE-p and HLs. It revealed 46 CpG sites in *HIF1AN *CpG island 47. **Table S1** Methylation distribution of CpG sites of *EGLN1* and *HIF1AN* in each subject of the three study groups i.e., HAPE-f, HAPE-p and HLs. **Table S2** Real-time PCR conditions for *EGLN1* and *HIF1AN*. **Table S3** Sodium bisulfite-conversion-based methylation PCR Primers and conditions for *EGLN1* and *HIF1AN*.

## Data Availability

The data presented in this study are available in the manuscript and in supplementary materials.

## Abbreviations

HA: High altitude
HAPE: High-altitude pulmonary edema
EGLN1: Egl nine homolog 1 (EGLN1)
PHD2: Prolyl hydroxylase domain protein 2
HIF1AN: Hypoxia inducible factor 1 subunit alpha inhibitor
FIH-1: Factor inhibiting HIF-1α
HAPE-p: HAPE-patients
HAPE-f: HAPE-free sojourners
HLs: Highland natives
SpO_2_: Peripheral blood oxygen saturation
OR: Odds ratio
SNPs: Single nucleotide polymorphisms
TF: Transcription factor
CI: Confidence interval

## References

[1] Fathollahipour S, Patil PS, Leipzig ND (2018). Oxygen regulation in development: lessons from embryogenesis towards tissue engineering. Cells Tissues Organs.

[2] Simon MC, Keith B (2008). The role of oxygen availability in embryonic development and stem cell function. Nat Rev Mol Cell Biol.

[3] An J, Rao A, Ko M (2017). TET family dioxygenases and DNA demethylation in stem cells and cancers. Exp Mol Med.

[4] Xu R, Sun Y, Chen Z, Yao Y, Ma G (2016). Hypoxic preconditioning inhibits hypoxia-induced apoptosis of cardiac progenitor cells via the PI3K/Akt-DNMT1-p53 pathway. Sci Rep.

[5] Bhatnagar A (2017). environmental determinants of cardiovascular disease. Circ Res.

[6] Hu CJ, Wang LY, Chodosh LA, Keith B, Simon MC (2003). Differential roles of hypoxia-inducible factor 1α (HIF-1α) and HIF-2α in hypoxic gene regulation. Mol Cell Biol.

[7] Fong GH, Takeda K (2008). Role and regulation of prolyl hydroxylase domain proteins. Cell Death Differ.

[8] Mishra A, Mohammad G, Norboo T, Newman JH, Pasha MQ (2015). Lungs at high-altitude: genomic insights into hypoxic responses. J Appl Physiol.

[9] Julian CG (2017). Epigenomics and human adaptation to high altitude. J Appl Physiol.

[10] D’Anna F, Van Dyck L, Xiong J, Zhao H, Berrens RV, Qian J, Bieniasz-Krzywiec P, Chandra V, Schoonjans L, Matthews J, De Smedt J, Minnoye L, Amorim R, Khorasanizadeh S, Yu Q, Zhao L, De Borre M, Savvides SN, Simon MC, Carmeliet P, Reik W, Rastinejad F, Mazzone M, Thienpont B, Lambrechts D (2020). DNA methylation repels binding of hypoxia-inducible transcription factors to maintain tumor immunotolerance. Genome Biol.

[11] Koslowski M, Luxemburger U, Türeci O, Sahin U (2011). Tumor-associated CpGdemethylation augments hypoxia-induced effects by positive autoregulation of HIF-1α. Oncogene.

[12] Kindrick JD, Mole DR (2020). Hypoxic regulation of gene transcription and chromatin: cause and effect. Int J Mol Sci.

[13] Moore LD, Le T, Fan G (2013). DNA methylation and its basic function. Neuropsychopharmacology.

[14] Fong G-H, Takeda K (2008). Role and regulation of prolyl hydroxylase domain proteins. Cell Death Differ.

[15] Lando D, Peet DJ, Gorman JJ, Whelan DA, Whitelaw ML, Bruick RK (2002). FIH-1 is an asparaginyl hydroxylase enzyme that regulates the transcriptional activity of hypoxia-inducible factor. Genes Dev.

[16] Sharma K, Mishra A, Singh HN, Prashar D, Alam P, Thinlas T, Mohammad G, Kukreti R, Syed MA, Pasha MAQ (2021). High-altitude pulmonary edema is aggravated by risk-loci and associated transcription factors in HIF-prolyl hydroxylases. Hum Mol Genet.

[17] Mishra A, Mohammad G, Thinlas T, Pasha MQ (2013). EGLN1 variants influence its expression and SaO2 levels to associate with high-altitude pulmonary edema and adaptation. Clin Sci.

[18] Luks AM, Auerbach PS, Freer L, Grissom CK, Keyes LE, McIntosh SE, Rodway GW, Schoene RB, Zafren K, Hackett PH (2019). Wilderness medical society practice guidelines for the prevention and treatment of acute altitude illness: 2019 update. Wilderness Environ Med.

[19] Mishra A, Kohli S, Dua S, Mohammad G, Thinlas T, Pasha MQ (2015). Genetic differences and aberrant methylation in the apelin system predict the risk of high altitude pulmonary edema. Proc Natl Acad Sci U S A.

[20] Bigham A, Bauchet M, Pinto D, Mao X, Akey JM, Mei R, Scherer SW, Julian CG, Wilson MJ, LópezHerráez D, Brutsaert T, Parra EJ, Moore LG, Shriver MD (2010). Identifying signatures of natural selection in Tibetan and Andean populations using dense genome scan data. PLoS Genet.

[21] Brutsaert TD, Kiyamu M, Elias Revollendo G, Isherwood JL, Lee FS, Rivera-Ch M, Leon-Velarde F, Ghosh S, Bigham AW (2019). Association of EGLN1 gene with high aerobic capacity of Peruvian Quechua at high altitude. Proc Natl Acad Sci U S A.

[22] Xiang K, Ouzhuluobu, Peng Y, Yang Z, Zhang X, Cui C, Zhang H, Li M, Zhang Y, Bianba, Gonggalanzi, Basang, Ciwangsangbu, Wu T, Chen H, Shi H, Qi X, Su B. Identification of a Tibetan-specific mutation in the hypoxic gene EGLN1 and its contribution to high-altitude adaptation. Mol Biol Evol. 2013;30(8):1889–98. 10.1093/molbev/mst090 23666208

[23] Lorenzo FR, Huff C, Myllymäki M, Olenchock B, Swierczek S, Tashi T, Gordeuk V, Wuren T, Ri-Li G, McClain DA, Khan TM, Koul PA, Guchhait P, Salama ME, Xing J, Semenza GL, Liberzon E, Wilson A, Simonson TS, Jorde LB, Kaelin WG, Koivunen P, Prchal JT (2014). A genetic mechanism for Tibetan high-altitude adaptation. Nat Genet.

[24] Brutsaert TD, Kiyamu M, Revollendo GE, Isherwood JL, Lee FS, Rivera-Ch M, Leon-Velarde F, Ghosh S, Bigham AW (2019). Association of EGLN1 gene with high aerobic capacity of Peruvian Quechua at high altitude. Proc Natl Acad Sci U S A.

[25] Zhi D, Aslibekyan S, Irvin MR, Claas SA, Borecki IB, Ordovas JM, Absher DM, Arnett DK (2013). SNPs located at CpG sites modulate genome-epigenome interaction. Epigenetics.

[26] Zhang X, Moen EL, Liu C, Mu W, Gamazon ER, Delaney SM, Wing C, Godley LA, Dolan ME, Zhang W (2014). Linking the genetic architecture of cytosine modifications with human complex traits. Hum Mol Genet.

[27] Childebayeva A, Jones TR, Goodrich JM, Velarde FL, Rivera-Chira M, Kiyamu M, Brutsaert TD, Dolinoy DC, Bigham AW (2019). LINE-1 and EPAS1 DNA methylation associations with high-altitude exposure. Epigenetics.

[28] Zhang R, Lai L, He J, Chen C, You D, Duan W, Dong X, Zhu Y, Lin L, Shen S, Guo Y, Su L, Shafer A, Moran S, Fleischer T, MoksnesBjaanæs M, Karlsson A, Planck M, Staaf J, Helland Å, Esteller M, Wei Y, Chen F, Christiani DC (2019). EGLN2 DNA methylation and expression interact with HIF1A to affect survival of early-stage NSCLC. Epigenetics.

