# Supplementary material for: Differential methylation in EGLN1 associates with blood oxygen saturation and plasma protein levels in high-altitude pulmonary edema
Source: Clin Epigenetics. 2022 Sep 30;14:123. doi: 10.1186/s13148-022-01338-z (PMC9526282; doi:10.1186/s13148-022-01338-z)
Supplement: Supplementary file 1 — Additional file 1. Fig. S1 Levels of SpO2 % in HAPE-p, HAPE-f and HLs. Fig. S2 Dot plot of CpG methylation in EGLN1 in the three study groups, i.e., HAPE-p, HLs and HAPE-f. It revealed 97 CpG sites in EGLN1 CpG island 179. Fig. S3 Dot plot of CpG methylation in HIF1AN in the three study groups, i.e., HAPE-f, HAPE-p and HLs. It revealed 46 CpG sites in HIF1AN CpG island 47. Table S1 Methylation distribution of CpG sites of EGLN1 and HIF1AN in each subject of the three study groups i.e., HAPE-f, HAPE-p and HLs. Table S2 Real-time PCR conditions for EGLN1 and HIF1AN. Table S3 Sodium bisulfite-conversion-based methylation PCR Primers and conditions for EGLN1 and HIF1AN. [file 13148_2022_1338_MOESM1_ESM.pdf]

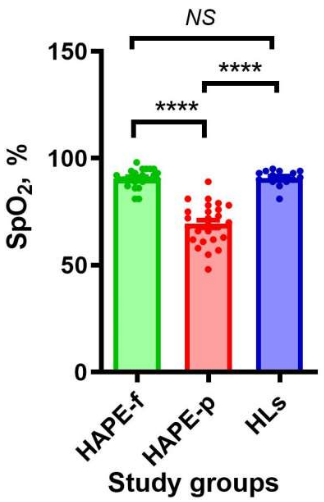

Supplementary Figure 1. Levels of SpO_2% in HAPE-p, HAPE-f and HLs.
SpO2: Blood arterial oxygen saturation level; NS: Not significant

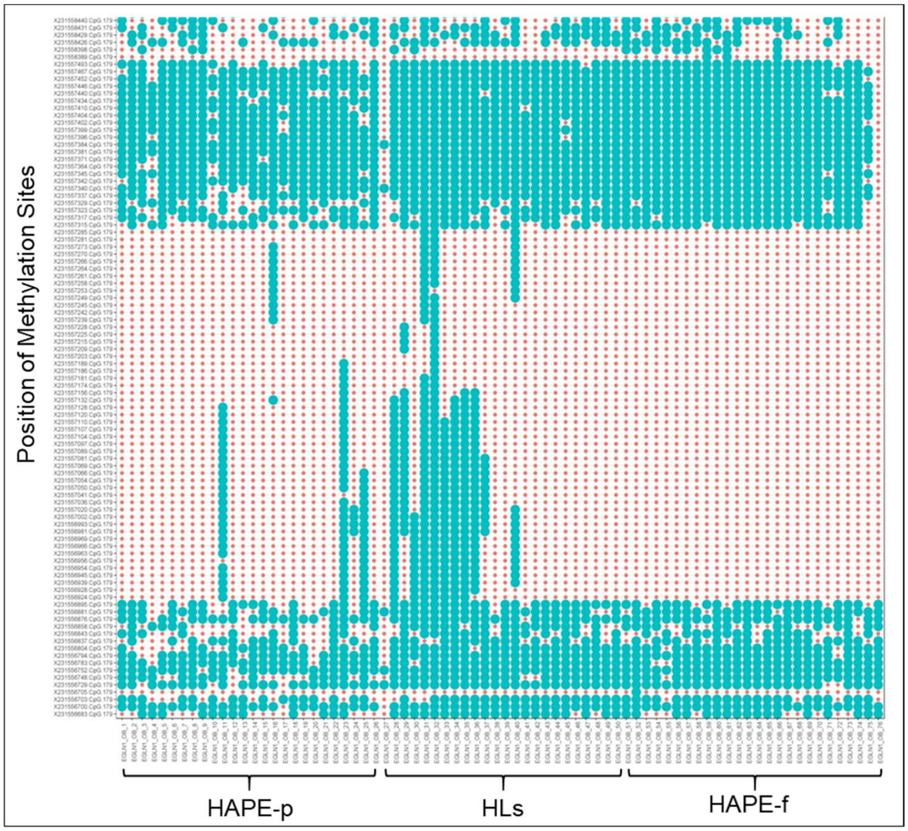

SupplementaryFigure 2. Dot plot of CpG methylation in *EGLN1* in the three study groups i.e. HAPE-p, HLs and HAPE-f. It revealed 97 CpG sites in *EGLN1* CpGisland 179.

• ,Un-methylated site; ● ,Methylated site.

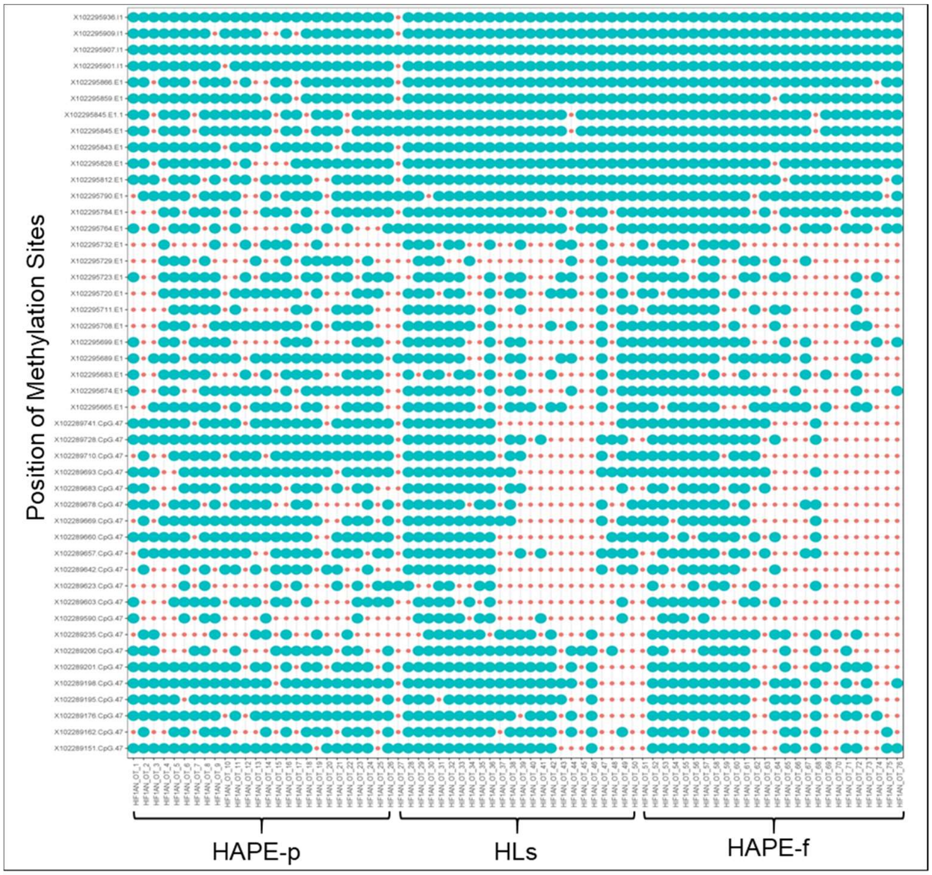

Supplementary Figure 3. Dot plot of CpG methylation in *HIF1AN* in the three study groups i.e. HAPE-f, HAPE-p and HLs. It revealed 46 CpG sitesin *HIF1AN*CpGisland 47.

• ,Un-methylated site; ●, Methylated site.

Supplementary Table 1. Methylation distribution of CpG sites of *EGLN1* and *HIF1AN* in each subject of the three study groups i.e., HAPE-f, HAPE-p and HLs.

	***EGLN1* 5mC sites**			***HIF1AN* 5mC sites**		
**Subjects**	**HAPE-f**	**HAPE-p**	**HLs**	**HAPE-f**	**HAPE-p**	**HLs**
1	42.27	36.08	5.15	65.22	67.39	17.39
2	42.27	39.18	65.98	93.48	65.22	86.96
3	38.14	35.05	67.01	93.48	52.17	93.48
4	40.21	19.59	50.52	86.96	69.57	95.65
5	37.11	35.05	87.63	93.48	82.61	93.48
6	41.24	34.02	93.81	84.78	82.61	95.65
7	43.30	35.05	67.01	95.65	67.39	93.48
8	39.18	40.21	71.13	95.65	86.96	82.61
9	41.24	38.14	71.13	71.74	86.96	73.91
10	44.33	23.71	70.10	80.43	54.35	95.65
11	41.24	58.76	53.61	80.43	76.09	50.00
12	43.30	31.96	34.02	58.70	63.04	73.91
13	40.21	26.80	40.21	45.65	65.22	73.91
14	37.11	29.90	64.95	52.17	67.39	50.00
15	40.21	31.96	31.96	56.52	63.04	52.17
16	44.33	46.39	36.08	43.48	84.78	54.35
17	42.27	23.71	40.21	45.65	69.57	45.65
18	36.08	35.05	41.24	60.87	82.61	47.83
19	38.14	31.96	40.21	36.96	73.91	32.61
20	36.08	29.90	39.18	32.61	52.17	47.83
21	32.99	28.87	40.21	41.30	67.39	69.57
22	35.05	40.21	40.21	58.70	76.09	36.96

23	38.14	70.10	40.21	43.48	84.78	78.26
24	36.08	36.08	41.24	32.61	84.78	65.22
25	27.84	47.42		30.43	65.22	
26	13.40	40.21		39.13	69.57	
**Mean**	38.14	36.36	51.37	62.29	71.57	66.94
**Std Dev**	6.3	10.6	20.1	22.7	10.6	23.0

Supplementary Table 2. Real-time PCR conditions for *EGLN1* and *HIF1AN*.

Gene	Primer sequence	Cycling conditions	Product size (bp)
EGLN1	F 5'- GCT ACA AGG TAC GCA ATA ACT G-3'<br>R 5'- TGG ATC AAA GGC TCT AGA AGA C -3'	I94°C 10', D94°C 15",<br>A60°C 60", 40 cy	154
HIF1AN	F 5'- TGA GTT CGT TGA GAA ACT GC -3'<br>R 5'- TGT CAC ATT TCC TTC CAT GC-3'	I94°C 10', D94°C 15",<br>A60°C 60", 40 cy	211

F: forward; R: reverse; I: initial denaturation; D: denaturation; A: annealing; cy: cycles.

Supplementary Table 3. Sodium bisulfite-conversion-based methylation PCR Primers and conditions for *EGLN1* and *HIF1AN*.

**Gene**<br>**Primers**	**Primer sequence**	**Cycling conditions**	**Product**<br>**(bp)**
EGLN1			
HP1F<br>HP1R	F5'-GGGAAAAAGGGTTATAGTTAAGTTATTT-3'<br>R5'-AACTCCCACATAACCAAAAACATATTC-3'	**I**95°C 5', **D**95°C 30", **A**58°C 30", **E**72°C 30", 38cy, **FE**72°C 10'	212
HP2F2<br>HP2R2	F5'-TTATTTTATTGATTTTGAGGGGTTA-3'<br>R5'-CACATAACCAAAAACATATTCCC-3'	**I**95°C 5', **D**95°C 30", **A**59.3°C 30", **E**72°C 30", 38cy, **FE**72°C 10'	244
HP3F3<br>HP3R3	F5'-TTTTTGGTTATGTGGGAGGTTAG-3'<br>R5'-TATCCAAAAAATAAAATAAAAAATC-3'	**I**95°C 5', **D**95°C 30", **A**52.6°C 30", **E**72°C 30", 38cy, **FE**72°C 10'	206
HP4F4<br>HP4R4	F5'-GGGATGTTTTTTGTAGTTATTGGATT-3'<br>R5'-AAACCTACCTAATTTTCCCTTCTTC-3'	**I**95°C 5', **D**95°C 30", **A**60°C 30", **E**72°C 30", 38cy, **FE**72°C 10'	260
HP5F5<br>HP5R5	F5'-TTTGTAGTTATTGGATTTTTAAGATTT<br>R5'-AAACCTACCTAATTTTCCCTTCTTC-3'	**I**95°C 5', **D**95°C 30", **A**59.3°C 30", **E**72°C 30", 38cy, **FE**72°C 10'	251
HP6F6<br>HP6R6	F5'-GTGGTTTTTGGTTTTGGAGAGT-3'<br>R5'-CCCTAATCTAAAACCCTACCAATCT-3'	**I**95°C 5', **D**95°C 30", **A**60°C 30", **E**72°C 30", 38cy, **FE**72°C 10'	259
HP7F7<br>HP7R7	F5'-TGGTTTTTGGTTTTGGAGAGTT-3'<br>R5'-CTTCCCATAAACCCTAATCTAAAAC-3'	**I**95°C 5', **D**95°C 30", **A**59.3°C 30", **E**72°C 30", 38cy, **FE**72°C 10'	269
HP8F8<br>HP8R8	F5'-TAGTGGAGTTTAGGATGTAGAGTTGATAT-3'<br>R5'-CAAAACCAAAAACCACAACCT-3'	**I**95°C 5', **D**95°C 30", **A**59.3°C 30", **E**72°C 30", 38cy, **FE**72°C 10'	250

HIF1AN			
EP1F1<br>EP1R1	F5’- TTTTTTTATTGTTGTAAGGAGTATTAG-3’<br>R5’- CTTAACCTTTACTTTTCCCTTAACC-3’	**I**95°C 5', **D**95°C 30", **A**60°C 30", **E**72°C 30", 38cy, **FE**72°C 10'	231
EP2F2<br>EP2R2	F5’-TTTTTTTTATTGTTGTAAGGAGTATTAG-3’<br>R5’-CTTAACCTTTACTTTTCCCTTAACC-3’	**I**95°C 5', **D**95°C 30", **A**56°C 30", **E**72°C 30", 38cy, **FE**72°C 10'	232
EP3F3<br>EP3R3	F5’- GGGAGTTTGATTTTTGGATTTAGT-3’<br>R5’-ATCATAACTACTCTTCCTCCAAACC-3’	**I**95°C 5', **D**95°C 30", **A**58°C 30", **E**72°C 30", 38cy, **FE**72°C 10'	176
EP4F4<br>EP4R4	F5’- GGGAGTTTGATTTTTGGATTTAGT-3’<br>R5’-AATCATAACTACTCTTCCTCCAAACC-3’	**I**95°C 5', **D**95°C 30", **A**60°C 30", **E**72°C 30", 38cy, **FE**72°C 10'	177
EP5F5<br>EP5R5	F5’- TGGGTTGTTTATGAGTAGTATGGA-3’<br>R5’-CTTCTCAACCTAAACAATTTCAATC-3’	**I**95°C 5', **D**95°C 30", **A**59°C 30", **E**72°C 30", 38cy, **FE**72°C 10'	171
EP6F6<br>EP6R6	F5’-TTGGGTTGTTTATGAGTAGTATGGA-3’<br>R5’-CTTCTCAACCTAAACAATTTCAATC-3’	**I**95°C 5', **D**95°C 30", **A**57°C 30", **E**72°C 30", 38cy, **FE**72°C 10'	172
EP7F7<br>EP7R7	F5’-TAGTTGGTTAGTTAGAAGAGTGATT-3’<br>R5’-TACTCATAAACAACCCAATAATTTC-3’	**I**95°C 5', **D**95°C 30", **A**59°C 30", **E**72°C 30", 38cy, **FE**72°C 10'	109
EP8F8<br>EP8R8	F5’-GTAGTTGGTTAGTTAGAAGAGTGATT-3’<br>R5’-TACTCATAAACAACCCAATAATTTC-3’	**I**95°C 5', **D**95°C 30", **A**60°C 30", **E**72°C 30", 38cy, **FE**72°C 10'	110
EP9F9<br>EP9R9	F5’-CGTATATTTCGCGTCGGTTTGGAG-3'<br>R5’-CTACGCTCCTCCGCACTCGAC-3'	**I**95°C 5', **D**95°C 30", **A**55°C 30", **E**72°C 30", 38cy, **FE**72°C 10'	291

EP10F<br>EP10R	F5'-TTCGTTTCGTTATTCGTTTGCGG-3'<br>R5'-CGAAACCGAACCTCGACTTCTAC-3'	**I**95°C 5', **D**95°C 30", **A**58.6°C 30", **E**72°C 30", 38cy, **FE**72°C 10'	222
EP11F<br>EP11R	F5'-GTAGAAGTCGAGGTTCGGTT-3'<br>R5'-ACCTATACGACGAATAACAACCTA-3'	**I**95°C 5', **D**95°C 30", **A**54.2°C 30", **E**72°C 30", 38cy, **FE**72°C 10'	308
EP12F<br>EP12R	F5'-AGGTTGTTATTCGTCGTATAGGTT-3'<br>R5'-ATTCTCCATCTTCCCGCACAA-3'	**I**95°C 5', **D**95°C 30", **A**51.7°C 30", **E**72°C 30", 38cy, **FE**72°C 10'	357
EP13F<br>EP13R	F5'-AGGGTTCGGCGGTGGTTGTC-3'<br>R5'-CGACACGATATACTCGAACGCCAA-3'	**I**95°C 5', **D**95°C 30", **A**60°C 30", **E**72°C 30", 38cy, **FE**72°C 10'	199
EP14F<br>EP14R	F5'-GAGTATATCGTGTCGTGTATGAA-3'<br>R5'-TAACCAACTACCCGTCCGTA-3'	**I**95°C 5', **D**95°C 30", **A**60°C 30", **E**72°C 30", 38cy, **FE**72°C 10'	138

F: forward; R: reverse; I: initial denaturation; D: denaturation; A: annealing; E: extension; FE: final extension; cy: cycles
